# Patients’ and Health Care Providers’ Opinions on a Supportive Health App During Breast Cancer Treatment: A Qualitative Evaluation

**DOI:** 10.2196/cancer.5334

**Published:** 2016-06-07

**Authors:** Danny A Young-Afat, Carla H van Gils, David J Bruinvels, Carmen C van der Pol, Arjen J Witkamp, Sieta Sijtsema, Yvette Jonasse, Rhodé M Bijlsma, Margreet G Ausems, Annelies M Bos, Desirée H van den Bongard, Helena M Verkooijen

**Affiliations:** ^1^ University Medical Center Utrecht Department of Clinical Epidemiology Julius Center for Health Sciences and Primary Care Utrecht Netherlands; ^2^ Px HealthCare Amsterdam Netherlands; ^3^ University Medical Center Utrecht Department of Surgery Utrecht Netherlands; ^4^ University Medical Center Utrecht Department of Plastic and Reconstructive Surgery Utrecht Netherlands; ^5^ University Medical Center Utrecht Department of Medical Oncology Utrecht Netherlands; ^6^ University Medical Center Utrecht Department of Medical Genetics Utrecht Netherlands; ^7^ University Medical Center Utrecht Department of Reproductive Medicine and Gynecology Utrecht Netherlands; ^8^ University Medical Center Utrecht Department of Radiation Oncology Utrecht Netherlands; ^9^ University Medical Center Utrecht Imaging Division Utrecht Netherlands

**Keywords:** breast cancer, health apps, quality of life, patient-reported outcomes, PROs

## Abstract

**Background:**

Health apps are increasingly being used in clinical care and may hold significant theoretical potential. However, they are often implemented in clinical care before any research has been done to confirm actual benefits for patients, physicians, and researchers.

**Objective:**

This study aimed to explore experiences of patients and health care providers with the use of a supportive breast cancer app during the first 6 months following diagnosis, in terms of benefits for clinical practice and research purposes.

**Methods:**

Between June 2013 and April 2014, breast cancer patients of all ages were invited shortly after diagnosis to use a supportive breast cancer app, and were followed for 6 months. Patients were asked to use the app at their own convenience. In-depth interviews were conducted regularly with patients and their medical team (ie, physicians and nurses) to evaluate their experiences.

**Results:**

A total of 15 patients aged 30-63 years participated. The medical team consisted of 7 physicians and 3 specialized breast cancer nurses. Out of the 15 patients, 12 (80%) used the app to obtain information on breast cancer and treatment. A total of 11 out of 12 patients (92%) evaluated this information as useful. All 15 patients used the app to record consultations with practitioners, and 14 (93%) found this useful. Symptom registration was used by 8 out of 15 patients (53%), and was found useful by 4 out of these 8 patients (50%). Overall, 14 out of 15 patients (93%) would recommend the app to other patients. The app, in particular the recording function, was rated as useful by 9 out of 10 medical professionals (90%), and they reported that it did not increase consultation time. These 9 professionals would recommend the app to their patients.

**Conclusions:**

This evaluation of a supportive health app shows positive experiences among patients and their medical teams. Based on experiences in this study, patients may need to be actively encouraged to regularly register symptoms within health apps to generate sufficient patient-reported app data for use in clinical practice and scientific research.

## Introduction

Health apps are increasingly being used by physicians and patients in routine clinical care [1]. Lancet Oncology predicted that by 2018, approximately 1.7 billion mobile phone and tablet users will have downloaded at least one health app [2]. These apps have the potential to be of benefit to patients, physicians, nurses, and researchers. The US Food and Drug Administration (FDA) has noted that health apps can help patients “in the management of their health and wellness, promote healthy living and gain access to useful information whenever and wherever they need it” [2]. Apple recently introduced ResearchKit, with the aim to combine patient data from various health apps and make them accessible to medical researchers [3]. This may further promote the use of health apps for research purposes.

In the field of breast cancer research, patient-reported outcomes (PROs) are becoming increasingly important to better understand and quantify symptoms, psychosocial well-being, and side effects of treatment from a patient’s perspective [4,5]. Mobile health apps may prove to be useful in the collection of PROs, as many patients already use their mobile phones to collect and share personal information. However, it is still unknown to what extent health apps can be used to collect reliable PROs.

The use of supportive health apps may hold significant theoretical potential, but little research has been done about actual benefits prior to implementation in clinical care [1,6-8]. This information should be available before physicians and nurses advise their patients to use an app during their treatment.

This study aimed to explore first experiences with the use of a supportive breast cancer app during diagnosis and treatment, with the aim to better understand potential benefits in clinical practice. In addition, we aimed to evaluate to what extent self-reported app data could be used for research purposes. The aim was to evaluate the app on three levels: patient experience and satisfaction, physicians’ and nurses’ opinions, and scientific potential.

## Methods

Between June 2013 and August 2013, and between March 2014 and April 2014, breast cancer patients consecutively visiting the Department of Surgery of the University Medical Center Utrecht in the Netherlands were invited to use a supportive breast cancer app. All patients were invited to participate, with the exception of patients who were unable to read and understand the Dutch language, patients under the age of 18 years, and patients who were considered too emotional to receive study information at the time of recruitment.

Shortly after diagnosis, the study was first introduced by a nurse practitioner and patients received written study information to read at home. If the patient was interested in participating, a meeting with the researcher was scheduled 1-3 days later for the informed consent procedure.

Patients were recruited within the first week after breast cancer diagnosis, which allowed them to start using the app prior to deciding on a final treatment plan. Each patient was followed for 6 months to evaluate her experiences with the app shortly after diagnosis, but also during treatment and after treatment was initiated. Patients were asked to use their own mobile devices. However, if they were interested in participating but did not have a mobile phone or tablet, the researcher offered an iPad, which they could borrow during study participation.

**Figure 1:**
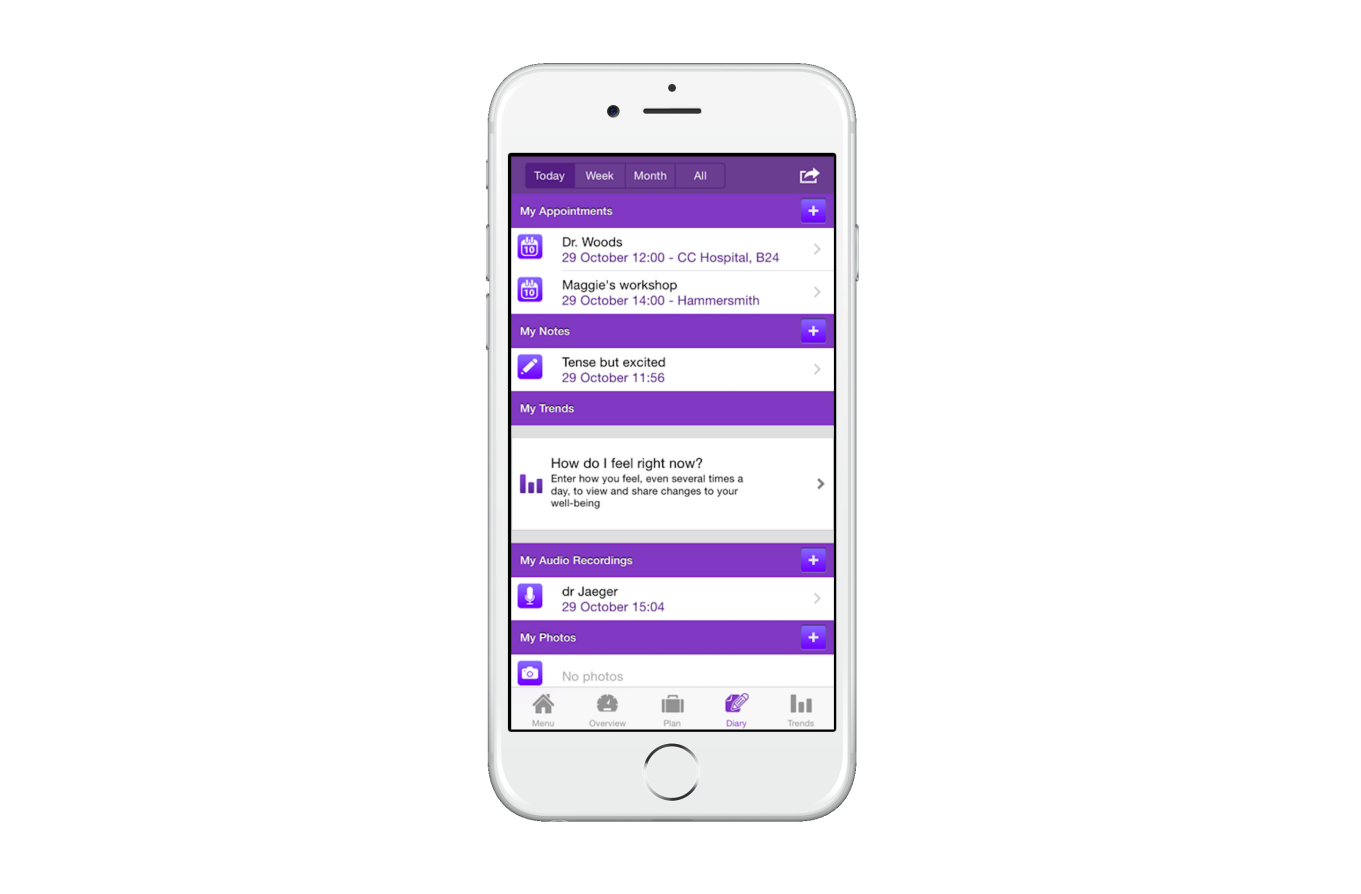
Screenshot of the OWise app (day overview).

**Figure 2:**
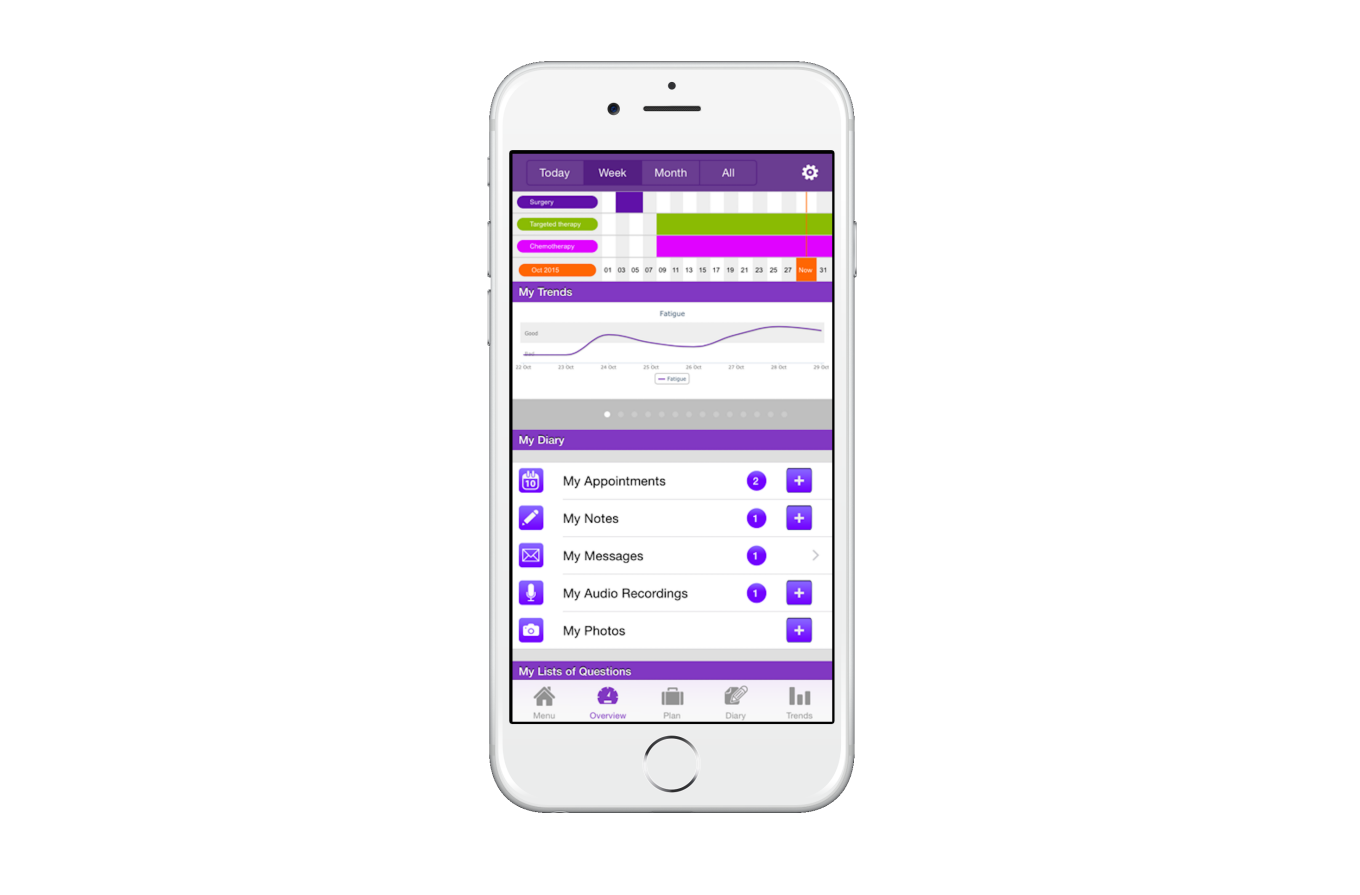
Screenshot of the OWise app (week overview).

Out of the few available Dutch supportive breast cancer apps, we chose to evaluate the OWise breast cancer app, version 1.0 (see Figures 1 and 2). This app was developed in 2013 by Px HealthCare, the Netherlands. We chose this app because it can be downloaded and used free of charge for iOS and Android platforms, and includes the following functionalities [9]:

1. Patient repository for information (eg, audio-recorded consultations and imaging).

2. Physical and psychological symptom registration (ie, pain, fatigue, mental mood, etc).

3. Timeline of treatment trajectory and appointments.

4. Personalized information about breast cancer and treatment according to Dutch breast cancer guidelines, tailored to tumor characteristics, age, and menopausal status.

A researcher briefly demonstrated these functions, after which patients were invited to use the app at their own convenience. There was no minimum amount of time to be spent using the app. This approach was chosen to understand which parts of the app patients would use based on their own needs.

In-depth interviews were conducted using a predefined, semistructured interview guide (see Multimedia Appendix 1). This interview guide was developed by our team of breast cancer physicians, specialized breast cancer nurses, and clinical epidemiologists, and it was based on questions that were considered relevant from a clinical point of view. All interviews were conducted by one researcher (DAYA) from the breast cancer research team of the University Medical Center Utrecht, who was not involved in the clinical care of the participants. Interviews with patients were conducted every 2 weeks in the first 3 months, and monthly in the last 3 months, either face to face or by phone. Nurses and physicians were interviewed once, shortly after they were first exposed to the app, and two times approximately 1 and 3 months after patients had used the app in their presence several times.

The interview guide was also designed to assess which app functions patients found most useful and for what reason. Questions for the medical team were designed to probe their opinions about the influence of the app on disease-related knowledge and disease-related behavior of patients during patients’ visits. In addition, medical professionals’ attitudes toward being recorded with the app were explored. The researcher interviewed each patient, physician, and nurse separately at all times. After each interview, a summary was transcribed and added to the participant's study file. Descriptive statistics were calculated using SPSS version 22 (IBM Corp) to summarize the data.

This study was approved by the Medical Ethics Committee of the University Medical Center Utrecht and was conducted according to the principles expressed in the Declaration of Helsinki. All patients gave written informed consent to participate in this study.

## Results

### Overview

During the recruitment period, 40 patients visited our medical center consecutively, of which 21 (53%) were not approached for participation because the nurse felt the setting was inappropriate for discussing studies (eg, too emotional) or because the patients were not interested in participating in any kind of research. A total of 19 patients received study information, after which 4 (21%) declined study participation; 1 patient declined because she would be treated in another hospital, 2 patients felt the interviews would take up too much time, and 1 patient was not interested in using the app.

### Patients’ Experiences

A total of 15 breast cancer patients with a mean age of 51 years (SD 10) participated in this study. The youngest patient was 30 years of age, while the oldest patient was 63 years of age. On average, each patient was interviewed eight times.

At baseline, patients were asked why they decided to participate in this study. The main reasons for participation were (1) an interest in this particular health app, (2) the hope of gaining benefit from using the app, (3) an interest in apps in general, and (4) an interest in participating in research to help future patients.

A total of 3 out of 15 patients (20%) expressed more specific reasons. One patient had received treatment for contralateral breast cancer in the past and was particularly interested in recording conversations with her medical team. During her previous treatment, she found it difficult to remember all the information provided by the various different physicians. Another patient had recently lost her husband to cancer and found it difficult, due to her current emotional mental state, to process and remember new information. She hoped that by having her treatment-related information and audio-recordings all in one place, she would be more in control. The third patient was a full-time, nonmedical researcher and found it interesting to be on the other end of a study for a change. Prior to entering the study, 10 patients out of 15 (67%) had frequently used apps on their mobile devices, while 5 (33%) were relatively inexperienced with the use of apps.

Personalized information on breast cancer and treatment as provided by the app was used by 12 out of 15 patients (80%). Out of the 12 patients who used this information, 11 patients (92%) found it useful (see Table 1). All patients (n=15) used the audio-recording function to record consultations with their nurses and physicians, and 14 (93%) of them found this to be useful. Overall, 14 out of 15 patients (93%) would recommend the app to other patients.

The patient who would not recommend the app to others reported that it did not add much to the information as provided by the medical team and on the Internet. She did not feel comfortable recording medical consultations and registering symptoms. Table 2 presents quotations regarding specific app functions as provided by patients.

**Table 1:** Patients’ and health care providers’ experiences with specific app functions.

Study participants	Characteristics and experiences with app		n (%)
**Patients**			
	**Age in years (n=15)**		
		30-39	3 (20)
		40-49	2 (13)
		50-59	8 (53)
		60-65	2 (13)
	Used information from the app		12/15 (80)
		Found it useful	11/12 (92)
	Used audio-recording function		15/15 (100)
		Found it useful	14/15 (93)
	Used symptom registration function		8/15 (53)
		Found it useful	4/8 (50)
	Would recommend app to other patients		14/15 (93)
**Physicians and nurses**			
	Found it useful for patients to record consultation		9/10 (90)
	Thought patients appeared to be better informed		2/10 (20)
	Would recommend this app to their patients		9/10 (90)

**Table 2:** Quotes from patients regarding specific app functions.

App functions	Supportive quotations (n=14)	Nonsupportive quotations (n=1)
Information about breast cancer and treatment (based on Dutch guidelines)	“A very useful overview of information, with links to all relevant websites in one place. I thought that was really helpful.”	“To me the information in the app does not add much to the information that I can find on the Internet or as provided by my doctors.”
Patient repository for information (eg, audio-recorded consultations)	“I shared the audio with my parents who could not be present at the consult. It was comforting to know that they heard the information firsthand from the surgeon instead of my own interpretation. At the same time, I heard important things during playback that I had missed during the initial consultation.” “I forgot important things the doctor said and it felt comforting to know that I could listen to the conversation again. From that moment on, I recorded every consult.” “I regretted not recording several important consultations, because the ones I did record I listened to several times.”	“I can imagine it being helpful to some patients, but I personally do not need to listen to a consult again. I would feel uncomfortable having to ask every doctor if it’s okay to record the conversation.”
Symptom and feeling registration	“I used the symptom registration function on a daily basis during the first month until 2 weeks after the surgery. It helped me a lot to see the graphical overview of my symptoms on a weekly basis. I stopped using it when I started to feel better and my symptoms did not fluctuate anymore.“ “The app gave me a familiar feeling in a difficult time of continuously changing faces and feelings, and registering my emotions in the app helped me to express feelings that I would have otherwise kept to myself.”	“I’m a very grounded person. Breast cancer happened to me, but I do not want to think about it daily. I’ve never kept a diary in my life, so I have no desire to start one now.”
Timeline	“In the timeline, I registered all my appointments. Keeping an overview of ongoing treatments was very difficult with so many different doctors and appointments, but the app helped me to keep that overview, which made me feel in control.”	“I already have a calendar for all my other personal appointments, so I do not need an app for this. I do not feel the need to separate personal appointments from hospital appointments. I’ll just deal with it all at the same time.”

### Physicians’ and Nurses’ Experiences

The medical breast cancer team consisted of 2 breast surgeons, a medical oncologist, a radiation oncologist, a plastic surgeon, a gynecologist, a clinical geneticist, and 3 specialized breast cancer nurses. All 10 team members were recorded by patients at least once, and they all reported that being recorded did not influence consultation time. A total of 2 physicians out of the 10 team members (20%) indicated that they chose their wording more carefully. These 2 physicians indicated that they felt uncomfortable while being recorded at first, but also that they got used to it over time. The audio-recording function was rated as useful by 9 out of 10 (90%) health care professionals. Of the 10 team members, 2 physicians (20%) had the impression that patients were better informed as a result of using the app. Overall, 9 out of 10 medical professionals (90%) would recommend the app to their patients. The 1 physician (10%) who would not recommend the app to patients believed the app did not add to the care and information as already provided by physicians and nurses (see Textbox 1).

**Quotes from health care providers about the app in clinical practice.**

“A patient, who was hesitant at first to record the consult, called my office to thank me for letting her record it. She and her husband heard important things during playback that both of them missed during our conversation.”

“I felt hesitant, and even a bit upset, while being recorded the first time. I noticed that I was paying closer attention to what I was saying. However, after a couple of times I did not notice the devices anymore and patients were so enthusiastic about it that I started to like it. I really think the app can be a very helpful tool, also for physicians.”

“A patient mentioned that she forgot when, and how, she would get the results of her test. Several days later she received a letter from the hospital, but she was too afraid to open it. She wanted to call the office to ask about the content of the letter, but it was off-hours. She then remembered that she had recorded the consult and found answers to her questions, after which her anxiety went away. In this case, it was simply the letter confirming the next appointment, that I luckily had mentioned during the recorded consult.”

“Personally, I don't think that health apps can add to the information we provide to our patients. We are able to provide patients with the information they need, when they need it, while also helping them understand what this medical information actually means.”

**Figure 3:**
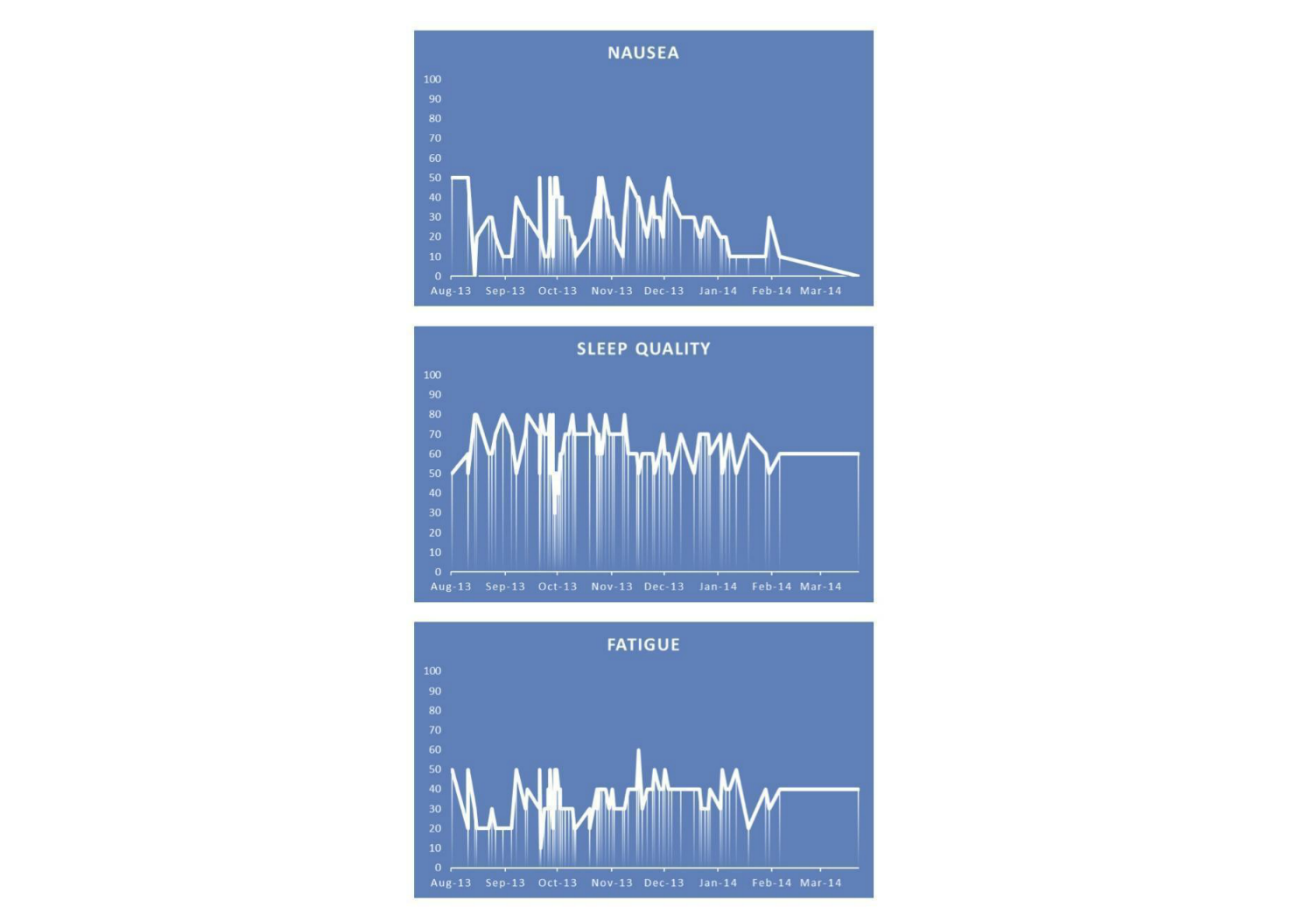
Example of a graphical overview of patient-reported outcomes as obtained from the OWise app's symptom registration function. The levels of nausea, sleep quality, and fatigue range from minimum (0) to maximum (100). A vertical line corresponds with the input of data by the patient. This patient received chemotherapy between August 2013 and December 2013. This data was provided by Px HealthCare with written permission from the patient.

### Scientific Potential of Patient-Reported App Data

Out of 15 patients, 8 (53%) used the symptom registration function at least once during the first month after diagnosis: 1 patient used this function daily (923 data entries), 4 patients weekly (121-355 data entries), and 3 patients monthly (10-30 data entries). Out of 15 patients, 7 (47%) never used this function. Out of the 8 patients who used this function, 4 (50%) found it useful. An example of symptoms registered by one patient is presented in Figure 3. This patient received neoadjuvant chemotherapy during the first 3 months of her treatment.

## Discussion

### Principal Findings

This study of a breast cancer support app shows positive experiences among patients and their medical team. The app functions patients found most useful were the option to record audio from consultations with their medical team, and the personalized information about disease and treatment. Physicians and nurses found the recording function most useful and would recommend the app to their patients.

Patients were asked to use the app at their own convenience, which made it possible to assess which functions of the app they wanted to use. This was based on their own needs in routine clinical practice. In this group of patients, the use of a symptom registration function varied from never to several times a day. With limited data entries in this small study group, we did not further explore the PROs that were generated from the app. We suggest that patients may need to be actively encouraged to regularly register their symptoms in the app. If this is done between hospital visits, results could then be shared during visits with their physicians and/or nurses. The medical team could then address symptoms that may have been left unnoticed otherwise, while researchers could evaluate these PROs in clinical studies when patients consent to the use of their data for research purposes.

The app in this study stores all audio-recordings on the mobile device, but, in contrast to the standard recording function on mobile devices, only allows for playback within the app without the option to edit or share the file with others. As a result, audio files are not stored on external servers or in Internet clouds, which serves as protection for patient data, but also protects the recorded physician/nurse against uncontrolled sharing and editing of their words [10]. This feature was appreciated by several members of our medical team and increased their willingness to be recorded. We recommend using apps that incorporate these kinds of conditions and restrictions, to allow audio-recording in the consulting room with protection of all parties involved in the recording process.

In this study, we chose to collect data by frequent in-depth interviews in order to obtain a complete first impression on the aspects of the app that patients and the medical team (dis)liked or found useful. The implication of this approach was that we could only include a small number of patients, which limits generalizability of the results. The strengths of this study were that we included patients of all ages, with or without an interest in apps, but also included a multidisciplinary medical team, which allowed for an in-depth evaluation of the needs of a relatively wide range of patients and medical professionals.

### Conclusions

This qualitative evaluation of a supportive breast cancer app shows benefits for patients and their medical teams, especially because of the option to make audio-recordings of consultations and the availability of relevant information in the app. However, in this study group, the use of the feature to register symptoms varied between patients. We recommend that future studies aiming to use patient-reported app data for scientific research encourage patients to regularly register their symptoms within these apps to generate sufficient data.

## Appendix

Multimedia Appendix 1 Semistructured interview guide.

## Abbreviations

FDA: US Food and Drug Administration
PRO: patient-reported outcome
