# Supplementary material for: Patients’ and Health Care Providers’ Opinions on a Supportive Health App During Breast Cancer Treatment: A Qualitative Evaluation
Source: JMIR Cancer. 2016 Jun 7;2(1):e8. doi: 10.2196/cancer.5334 (PMC5369627; doi:10.2196/cancer.5334)
Supplement: Multimedia Appendix 1 [file cancer_v2i1e8_app1.pdf]

**Multimedia Appendix 3: Semistructured interview guide**

All questions are used as guidance during interviews with patients. Questions are used to trigger response and follow-up questions are conceived spontaneously. After each answer follow-up questions are asked to understand why something was used/helpful, and why not.

**Questions for patients:**

- How often did you use the OWise app in the past 2 weeks/month? How often since the start of the study?
- Did you use the app to prepare for conversations with your doctors and nurses? Did you use information from the app?
- Did you record any conversations with your doctors or nurses? If so, have you listened to those recorded conversations again?
- Did you register symptoms/mood within the app?
- What is your overall opinion about the app? Does using the app help you in any way during this time of your life/treatment?
- Do you think the app is helpful when preparing for conversations with your doctors?
- Would you recommend the app to other patients?

**Question for doctors and nurses:**

- Do you think the app has an effect on your conversations with patients? Do you, or do they, behave differently?
- Do patients appear to be better informed?
- Do patients use the recording function during conversations with you? How did you feel about being recorded? Did you behave differently?
- Would you recommend this app to your patients?
